# Goal-dependent current compensation and drift in surf scoter flocks

**DOI:** 10.1186/s40462-016-0068-7

**Published:** 2016-01-31

**Authors:** Ryan Lukeman, Alexis Christie, Ronald C. Ydenberg

**Affiliations:** Department of Mathematics, Statistics, and Computer Science, St. Francis Xavier University, P.O. Box 5000, Antigonish, NS Canada; Department of Biological Sciences, Simon Fraser University, 8888 University Drive, Burnaby, BC Canada

**Keywords:** Current compensation, Drift, Flocking, Collective motion

## Abstract

**Background:**

Animals moving through air or water toward a goal frequently must contend with fluid currents, which can drift the actual path of the animal away from the direction of heading. Whether, and to what degree, animals compensate for currents depends on the species and environmental context, but plays an important role in the movement ecology of the species. In this paper, flocks of surf scoters (*Melanitta perspicillata*), an aquatic diving duck, were individually tracked during collective foraging in the presence of sideward water currents to assess the individual compensatory response while moving from open water toward the foraging location versus return to open water.

**Results:**

During short-range movement toward the foraging location, surf scoters moved more slowly, and compensated for currents by orienting diagonally into the current to maintain a perpendicular track to the goal. In contrast, during return to open water, surf scoters moved faster, and maintained a perpendicular orientation away from the foraging location, and allowed the sideward current to drift their track diagonally.

**Conclusions:**

Surf scoters show a behavioural flexibility in response to currents, alternately using compensation and drift as the movement goal and consequent cost of accuracy change.

## Background

Any organism travelling through moving air or water toward a goal must account for the fact that its actual track will be shifted from its heading (i.e. the direction it is pointing). This is called ‘drift’, and has been studied extensively among diverse taxa of long-distant migrants [1, 2], who must compensate for winds or currents to reach their goal. Drift compensation also occurs over much smaller scales. For example, common eiders (*Somateria mollissima sedentaria*) diving in polynyas with strong tidal currents descend at an angle to avoid being swept under the sea ice [3]. The Costa Rican fish *Brycon guatemalensis* lives in fast-flowing streams and consumes fruits that fall from overhanging trees. They accelerate toward the point of impact before a falling fruit has even hit the stream surface, adjusting their heading to take the current into account [4]. Stream fish such as Arctic grayling (*Thymallus arcticus*) must take account not only of the current in setting a course to intercept prey items, but take into account that the prey item is also moving [5].

The context for most studies of drift compensation is long-distance movement, which usually has reaching a particular destination as its goal. Other behavioral contexts such as foraging, or avoiding danger have different goals, but have been little studied. The common eiders described above must reach the benthos to find prey, but must especially avoid being carried out of the polynya. The fruit-catching fish of Costa Rica must minimize the interception time, as they compete in groups and the first individual there obtains the fruit. These considerations modify the purpose of drift compensation mechanisms beyond merely hitting the target. The particular ecology of each situation may thus require different or additional mechanisms to compensate for drift, with drift compensation itself forming part of the overall suite of tactical adjustments made to reach the objective (e.g. maximize intake rate). Krupczynski & Schuster [4] assert that the study of such ‘local’ phenomena could expand our knowledge of compensatory mechanisms.

We describe the movements of a group of surf scoters (*Melanitta perspicillata*) to and from a foraging site across an open stretch of water in which a current was flowing. Surf scoters are seaducks, and dive to obtain their food, swallowing prey such as clams and mussels whole and crushing them in their muscular gizzards. During winter they are highly gregarious, and groups exhibit co-ordinated movements as well as synchronous diving [6, 7]. In the situation described here, surf scoters moved toward a food site (a piling) where they dove and captured mussels before swimming away again to rest for a time across the water body.

## Methods

**Fig. 1:**
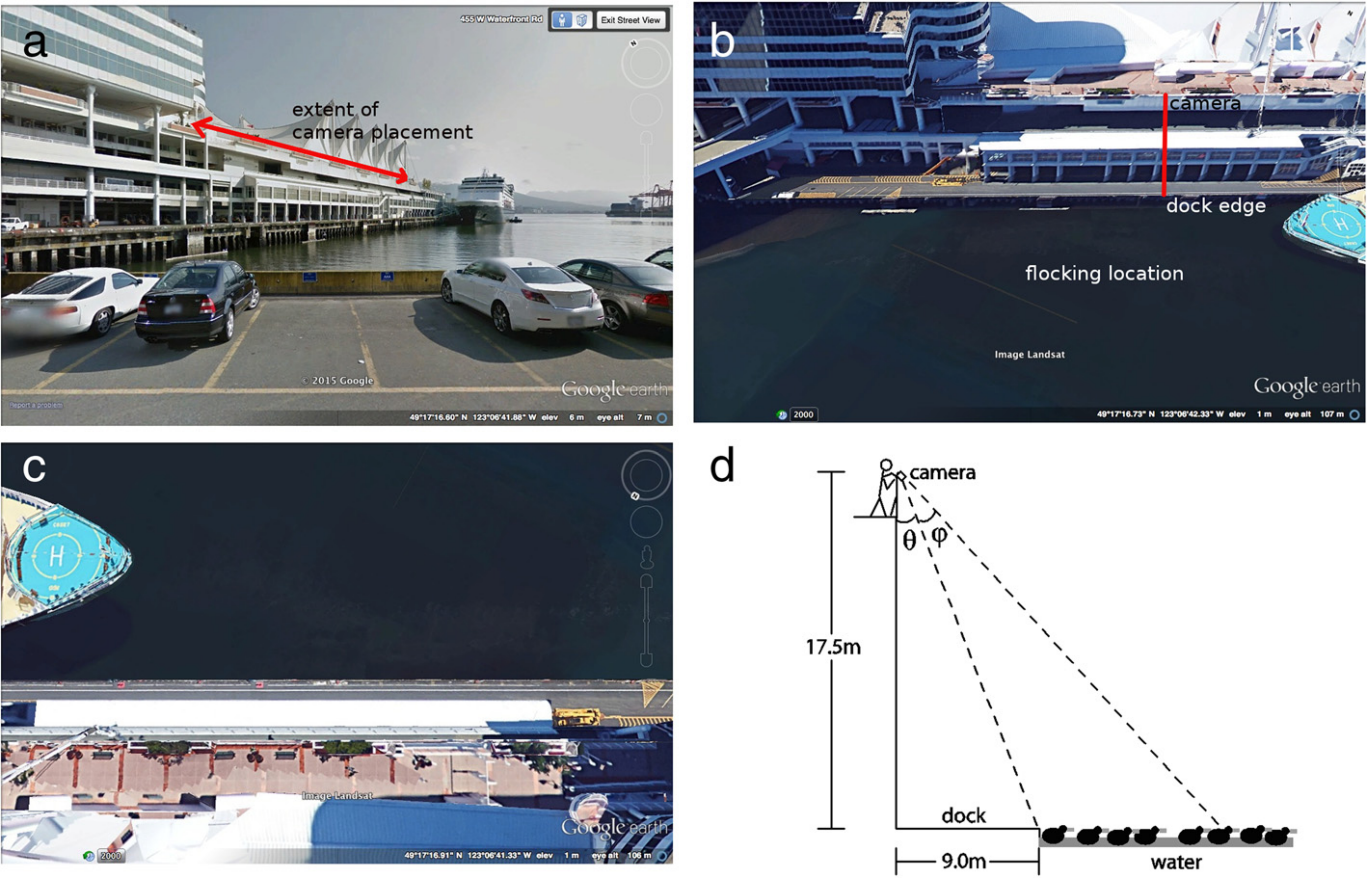
Location of field study: Canada Place, Vancouver British Columbia. The camera with tripod was placed along a promenade ledge, to be aligned with approaching scoters (**a**), where extent of camera placement is shown in red. Collective motion in the flocking location (**b**) was recorded from the overhead location (top view in (**c**)). A schematic diagram of the experimental setup is shown in (**d**)

Data were gathered between March 1 and March 12, 2008, by time-series photography, taken from a public promenade at Canada Place, in downtown Vancouver, British Columbia. The promenade is elevated, and overlooks the waters of Burrard Inlet, where overwintering surf scoters gathered for collective foraging on the surface of the water (Fig. 1). A Nikon D70S DSLR camera was used with a Nikon AF-S Nikkor 18–70 mm ED lens, fixed at maximal focal length (70 mm), and attached to a tripod. Photographs of surf scoters collectively swimming on the water surface were taken in continuous autofocus mode at a rate of three frames per second (fps) at a resolution of 1000 × 1504 pixels, with an aperture setting of f4.5. Exposure times ranged from 1/8000–1/250 s.

By aligning the lower edge of the camera viewfinder with the edge of the stationary dock below the promenade, the oblique overhead angle was fixed and quantified. Images were then taken in succession from a fixed elevation as soon as individuals entered the frame, and continued until individuals left the frame. In this way, time-series of collective movements were recorded. Motion was predominantly either toward the dock area (approaching the foraging location), or away from the dock area (toward open water) following a foraging bout; series were subcategorized into one of these two cases, and any series which did not clearly feature one of these two directed motions were discarded. Within each series, number of individuals ranged from a few tens to a few hundred.

Raw camera images underwent a vertical transformation (for camera axis angle) and horizontal transformation (projective perspective distortion: parallel lines in reality will appear to converge in an image taken at a non-zero camera axis angle) to convert image positions to real positions. Data was stored in JPEG images, and were processed in MATLAB using custom-built image processing routines to extract a set of individual positions in each frame. The positions were then linked in time using the ‘Particle Tracker’ plugin for ImageJ, in conjunction with custom-built tracking software in MATLAB. A detailed description of experimental methods and image analysis can be found in the supplementary material of [8].

In all, 14 separate time-series of approaches to the foraging area (‘approach’), and 14 of movement away from the foraging area, to open water (‘return’) were retained for analysis. Because individual trajectories were highly correlated (and thus not independent) both in space and time (groups moved in a highly-polarized fashion with little variation in group direction), velocity data for each series was averaged over all individuals and in time to obtain a single mean velocity vector characterizing the group motion for each of the 28 series. We define (*x*,*y*)−axes such that left-to-right corresponds to the positive *x* direction, bottom-to-top (away from the dock) corresponds to the positive *y* direction. The duration of each of the 28 series ranged from 10–45 s (30–135 frames).

Water currents were estimated for each time-series by using surf scoter excreta as intrinsic fluid tracers. For each series, a number of distinct tracers were identified. Centers-of-mass were calculated through successive frames, and current was estimated based on these tracks. Finally, each estimation from distinct tracers was averaged to generate a single current vector associated with each time series. Occasionally, if present, small pieces of floating debris were tracked and used as a secondary validation of calculated currents. Current was not measured directly by instruments in the water due to access limitations for security reasons, under control of Canada Border Services Agency.

**Fig. 2:**
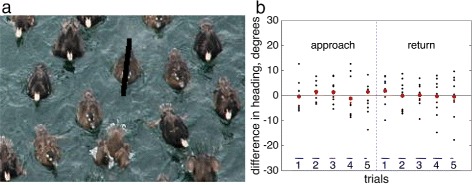
**a** Heading **H** is approximated from image data by manually drawing a straight-line approximation (*black line*) over the body of individuals. In **b**, the angle between **H**/||**H**|| and (**T** - **C**)/||**T** - **C**|| is shown for the 10 individuals across 5 sample time-series for approach and return (*black dots*), with averages in red. Current direction vectors for each of the sample trials are plotted in blue, and are to relative scale in direction and magnitude

In the data images, currents also appear indirectly via the difference in heading and track of individuals, From the data, the velocity relative to a stationary observer (track, **T**) can be measured. If the current vector **C** is known, the animal heading **H** can be calculated via **H**=**T**−**C**. Alternatively, if heading direction is calculated directly from individuals in data images, this can be used together with track **T** to verify **C**. Five randomly chosen time-series were chosen for each of the approach and return cases, and the middle frame analysed for each series by manually marking a straight-line approximation of heading direction on 10 individuals in an image frame in MATLAB (Fig. 2a). For each individual, the unit vector (**T**−**C**)/||**T**−**C**|| was computed. The angular difference between this expected heading direction, and the manual approximation value was then calculated. Results show relatively small error with no clear bias toward overestimation or underestimation of current (mean error is near zero degrees for each case). Standard deviation of angular error across the 10 trials ranged from 3.5–8.1 degrees. Results of this current validation are shown in Fig. 2b, where additionally, relative current direction and magnitude are plotted for each of the 10 trials. This close comparison between manually measured heading and computed heading via current and track mirrors results from a previous work drawn from the same data (see supplementary material of [8] for details).

## Results and discussion

Surf scoter flocks were typically cohesive and highly polarized when moving [8, 9], and displayed a high level of synchrony during foraging dives. A typical dive cycle for the flock included time spent in open water, followed by collective surface-swimming toward the dock area, where the flock then dove under the water surface with a high degree of synchrony to forage on mussels (*Mytilus trossulus*) attached to the pilings. Following the foraging bout, the flock resurfaced, again with high synchrony, and returned to open water, whereafter the cycle would repeat.

**Fig. 3:**
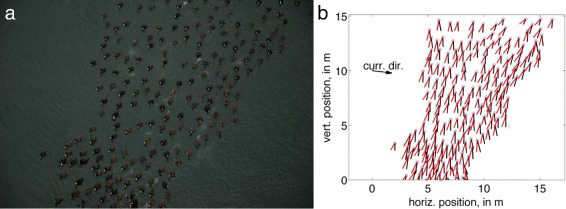
A snapshot of scoter flock approaching foraging grounds. Raw image from camera is shown in (**a**) showing highly polarized, cohesive motion and also excreta used as an intrinsic fluid tracer. Data extracted from (**a**) is shown in (**b**) after image analysis, transformation, and current calculation. In (**b**), individual velocity (track) vectors shown in black, with heading vectors shown in red. Current direction vector is not to scale relative to velocity vectors

**Fig. 4:**
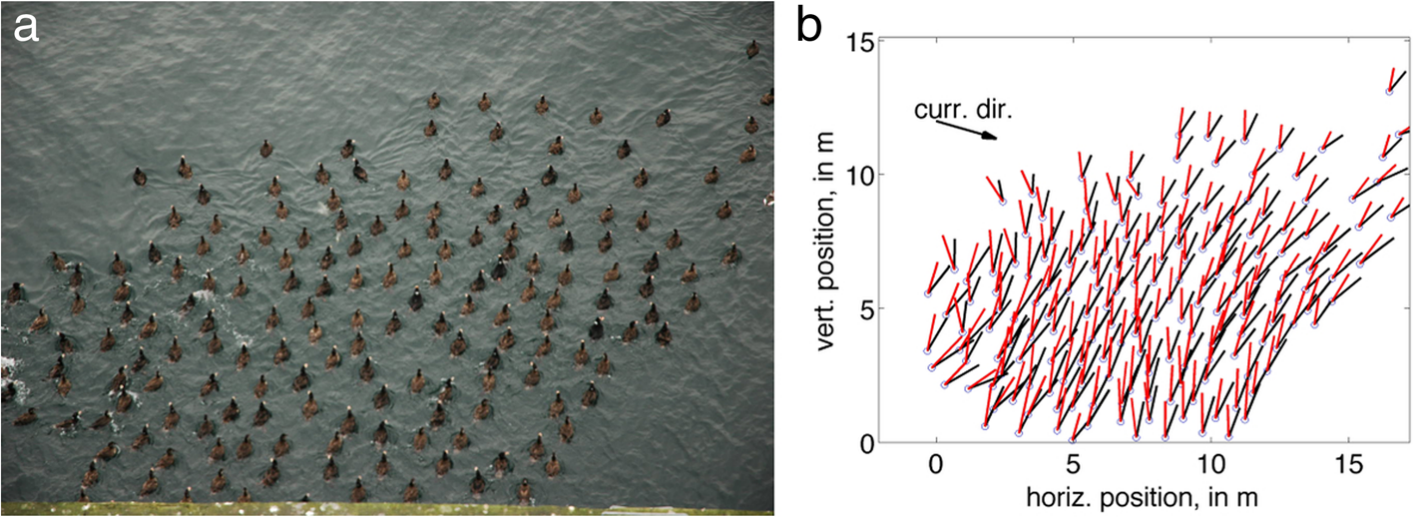
A snapshot of scoter flock returning from foraging grounds, in raw form (**a**) and after processing (**b**) as in Fig. 2

Figures 3 and 4 show example snapshots of a surf scoter flock approaching, and returning from, the foraging location, respectively, together with digitized representations of the flock.

### Water currents

Surface-swimming during approach to, and return from the foraging area occurred in the presence of predominantly lateral water currents of varying strength, but always with positive *x* component: currents always moved from left to right in the frame in the data analyzed. Mean current velocity $\bar {\mathbf {C}}$ across all 28 series was (0.34,−0.05) m/s, (SD of *x*-component: 0.12 m/s, of *y*-component: 0.05 m/s). Mean current speed was 0.34 m/s (SD = 0.12 m/s).

### Speeds of motion

Mean speed of motion for individuals during approach to the foraging site was 0.90 m/s (SD = 0.14 m/s) significantly less than speed of return, 1.26 m/s (SD = 0.17 m/s). (*t*-test, *t*=−5.972,*p*<0.001). Removing the effect of water currents, the speed relative to water (‘waterspeed’) during approach increased slightly (0.956 m/s, SD = 0.109), and for return, decreased slightly (1.24 m/s, SD = 0.15 m/s). However, the approach waterspeed was still significantly slower (*t*-test, *t*=−5.530,*p*<0.001). Because the mean vertical component of the current was negative (toward the forage location), individuals moving to the forage location had an increase in vertical component of velocity, whereas returning individuals had a decrease. This was offset by the tendency of approaching individuals to orient horizontally against the current, whereas this tendency was not present in returning individuals (see below).

### Collective measures

Group polarization $\frac {1}{N}\sum _{i=1}^{N} \vec {u}_{i}$, where $\vec {u}_{i}$ is the unit velocity vector of individual *i* at time *t*, measures the degree to which the entire group is aligned with one another. Polarization values range from 0 (highly disordered) to 1 (perfectly aligned). During approach to the foraging site, flocks were highly aligned, with mean polarization across time-series of 0.96 (SD = 0.031). Polarization during return was 0.90 (SD = 0.077), significantly less than during approach (*t*=2.53, *p*=0.0218,), indicating that collectively, individuals align more strongly during approach than return. Mean nearest neighbour distance (NND), measuring the degree of density of the flock, when averaged across trials, was not significantly different (*p*=0.15) during approach (NND = 0.73 m, SD = 0.051 m) and return (NND = 0.704 m, SD = 0.071).

### Angle of approach and return

During approach, mean velocity (track) was (−0.10,−0.84) m/s, and mean heading was (−0.386,−0.80) m/s. Returning individuals had a mean velocity of (0.44,1.027) m/s and mean heading of (0.06,1.09) m/s. Thus, during approach, individuals tend to approach in a nearly perpendicular track relative to the dock, and orient against the current to accomplish this direction of approach. Conversely, returning individuals orient in a nearly perpendicular heading relative to the dock, but are drifted by the current, resulting in a more diagonal track away from the dock area.

To investigate the difference between direction of approach and return further, angles of approach and return were calculated, relative to the perpendicular direction of motion relative to the dock. Angles are measured positively in a clockwise direction from the perpendicular. The statistical analysis of angles is accomplished using circular statistics.

Individuals had a mean velocity approach angle of 8.37° (SD = 13.52°), and a mean velocity return angle of 20.62° (SD = 15.76°). The mean velocity return angle was significantly larger than in approach (one-sided Watson-William test, *F*=4.522,*p*=0.021). Conversely, individuals had a mean heading approach angle of 19.41° (SD = 17.21°), and a mean heading return angle of 8.67° (SD = 12.34°). The mean heading return angle was significantly smaller than in approach (one-sided Watson-William test, *F*=3.30,*p*=0.040).

**Fig. 5:**
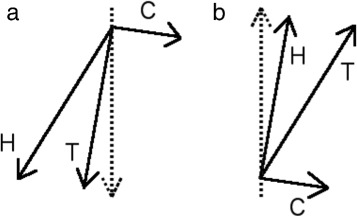
Schematic diagram of heading (H), track (T), and current (C) for (**a**) approach and (**b**) return from forage location. In approach, track is nearer than heading to the perpendicular line of approach (dotted), whereas in the return case, the opposite is true: heading is closer to perpendicular than track

A schematic diagram representing mean track and heading for approach (a) and return (b) is shown in Fig. 5.

### Approach and return: dependence on current

If individuals modulate heading to compensate completely for currents, we expect that as the lateral component of the current increases, so (oppositely) will the lateral component of the heading of the individual (assuming that the straight-line perpendicular is the ‘desired’ approach). Under perfect compensation, the regression line would have a slope of −1. Additionally, the horizontal component of the track should be uncorrelated with lateral current strength, since heading is adjusted to maintain a fixed track to the goal.

Conversely, should individuals be completely drifted by currents, headings would be uncorrelated with lateral current, while the horizontal component of the track will increase with the lateral current strength (with linear slope of 1, under perfect drift).

This analysis is less straightforward in the current study: the ‘goal’, whether approaching or returning from the forage location, is not necessarily the perpendicular route (e.g., an approaching group may be ‘aiming’ for a location in a direction not perpendicular to the dock, or a returning subgroup may be joining another group in open water which is at some non-zero angle to the surfacing location). Such varying environmental contexts will tend to reduce correlations that signify compensation or drift relative to a fixed direction of travel to a goal, though significant correlation may still be present.

**Fig. 6:**
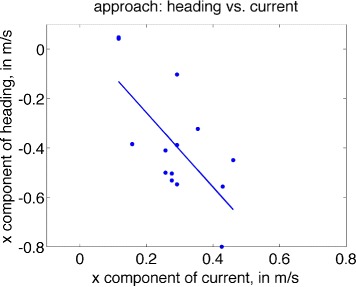
Scatterplot of average heading (lateral component) versus average lateral current, for each of 14 series featuring approach to the forage site, overlaid with the least-squares regression line

In Fig. 6, lateral components of heading and current are plotted for each series featuring group approach to the foraging area, together with a line of best fit. Lateral heading is significantly negatively correlated with lateral current (*r*=−0.684,*p*=0.007). The regression line is given by *h*_*x*_=0.044−1.51*c*_*x*_, with 95 % CI of (−2.516,−0.496) for the regression slope, providing evidence for an increased lateral component of heading during approach in response to stronger lateral current. Conversely, track was not strongly correlated with current during approach (*r*=0.30,*p*=0.296), also consistent with compensation.

**Fig. 7:**
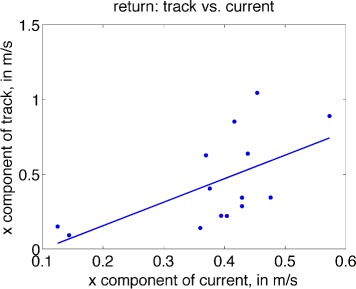
Scatterplot of average track (lateral component) versus average lateral current, for each of 14 series featuring return from the forage site, overlaid with the least-squares regression line

In Fig. 7, lateral components of track and current are plotted for each series featuring group return from the foraging site. Lateral track is significantly positively correlated with lateral current (*r*=0.603,*p*=0.0224). The regression line is given by *t*_*x*_=−0.158+1.572*c*_*x*_ with 95 % CI for regression slope given by (0.264, 2.88). Therefore, during return, increasing lateral current corresponds to an increase in lateral component of velocity, providing evidence that individuals are drifted by lateral currents. When comparing heading and current during return, no significant correlation with lateral current is found (*r*=0.265,*p*=0.3595), also consistent with a drift effect.

## Conclusions

The data show that the behavior of flocks during approaches and retreats differed in two ways. First, the swim ‘ground’ speed (i.e. relative to pilings) was about 30 % slower on approach than on retreat (0.90 vs. 1.26 m/s). Second, scoters adjusted their heading into the current on approach so as to maintain a track nearly perpendicular to the piling. On retreat they did not head into the current (see Fig. 5) and swam rapidly away from the piling. The data thus show that surf scoters compensate for drift induced by a lateral current during their approach to the foraging site, but not on the return to deeper water. The surf scoter flocks observed in this study were feeding on mussels (*Mytilus trossulus*) growing on pilings underneath the deck from which the observations were made. Mussels densely matted all the pilings, but as described by [10]), the communal defense of strongly binding byssal threads [11] limits the availability of mussels to scoters to openings or tears in the mat. Working along such breaches, surf scoter flocks can strip entire areas of the intertidal clean of mussels in days or weeks. Like most of the shellfish consumed by surf scoters, mussels are bulky prey, which means that they are often ingested at a rate that exceeds the processing ability of the digestive system. Consequently, surf scoters feed in bouts of intense diving over intertidal areas where their prey are found, alternating with periods of rest during which the flock moves out over deeper water while prey are processed. The approaches to highly specific targets and retreats to deeper water measured here therefore form part of their normal foraging routine. The presence of a tidal current flowing directly across their swim direction must also be a regular feature of their foraging environment. These observations imply that the specific, differing drift compensations observed on approach and return serve different functions. The straight-line, slow track suggests a cautious approach to a target location (necessarily one of the pilings, as this is where mussels grow). During retreat, individuals leave the foraging site much more quickly than they approached. They do not orient against the prevailing current to maintain a track identical to their approach, but use straight-line heading and allow themselves to be drifted by the current. The ‘goal’ or target while retreating is the region of deeper water well away from the surrounding structures (lower part of image in Fig. 1b), far larger and less specific than when approaching, so the costs of drifting are reduced. In fact, their choice of heading leads to the largest (vertical component of) distance covered from the dock for a given amount of energy expenditure, suggesting that, as might be expected of an animal facing a processing constraint [12] foraging surf scoters are efficiency maximizers.
